# Artificial Intelligence for Neonatal and Perinatal Mortality Prevention: A Systematic Review of Machine Learning and Deep Learning Applications

**DOI:** 10.3390/healthcare14152339

**Published:** 2026-08-01

**Authors:** Emmanuel Gutiérrez Jiménez, José Duván Márquez Díaz

**Affiliations:** System Engineering Department, Universidad del Norte, Barranquilla 081007, Colombia; jmarquez@uninorte.edu.co

**Keywords:** artificial intelligence, machine learning, deep learning, neonatal mortality, perinatal mortality, preterm birth, predictive models, systematic review, clinical decision support, low- and middle-income countries

## Abstract

**Background/Objectives:** Maternal, perinatal, and neonatal mortality remain major global health challenges, causing approximately 2.5 million neonatal deaths annually, particularly in low- and middle-income countries (LMICs). Although Artificial Intelligence (AI), including Machine Learning (ML) and Deep Learning (DL), is increasingly used to support healthcare decision-making, a comprehensive synthesis of its application to prevent prenatal, preterm birth, and neonatal deaths is lacking. This study systematically reviews the current state of research in this field. **Methods:** A Structured Literature Review (SLR) was conducted following a combined methodological framework integrating Massaro’s protocol and PRISMA 2020 guidelines. Searches were performed in Scopus, IEEE Xplore, and Google Scholar using domain-specific keywords. From 459 identified publications, 46 peer-reviewed studies published between 2018 and 2024 were selected through a four-step filtering and quality assessment process. Bibliometric and thematic analyses were performed. **Results:** ML techniques accounted for 71.7% of the selected studies, whereas DL approaches represented 28.3%. Neonatal death prediction was the most frequently investigated outcome (34.7% of publications). Most studies originated from Europe (39.1%) and North America (30.4%), while research from Latin America and Sub-Saharan Africa was scarce despite the high mortality burden in these regions. Key barriers included non-standardized clinical records, limited interoperability of health information systems, and the underrepresentation of LMIC populations in training datasets. **Conclusions:** AI shows significant potential for reducing maternal and neonatal mortality through predictive analytics. However, important geographical and methodological gaps remain. Future research should prioritize inclusive datasets and predictive frameworks adapted to resource-constrained healthcare settings.

## 1. Introduction

Neonatal mortality remains one of the most pressing challenges in global public health. Each year, approximately 2.4 million newborns die within the first 28 days of life, accounting for nearly 47% of all deaths in children under five [1,2]. The United Nations 2030 Agenda addresses this crisis through the Sustainable Development Goals (SDGs) [3], establishing under SDG 3 (Good Health and Well-Being) the target of reducing neonatal mortality to at least 12 deaths per 1000 live births in all countries by 2030 [4]. Despite global progress, this target remains unmet in large segments of low- and middle-income countries, where structural barriers in health systems continue to hinder timely clinical intervention [1,4].

**Figure 1:**
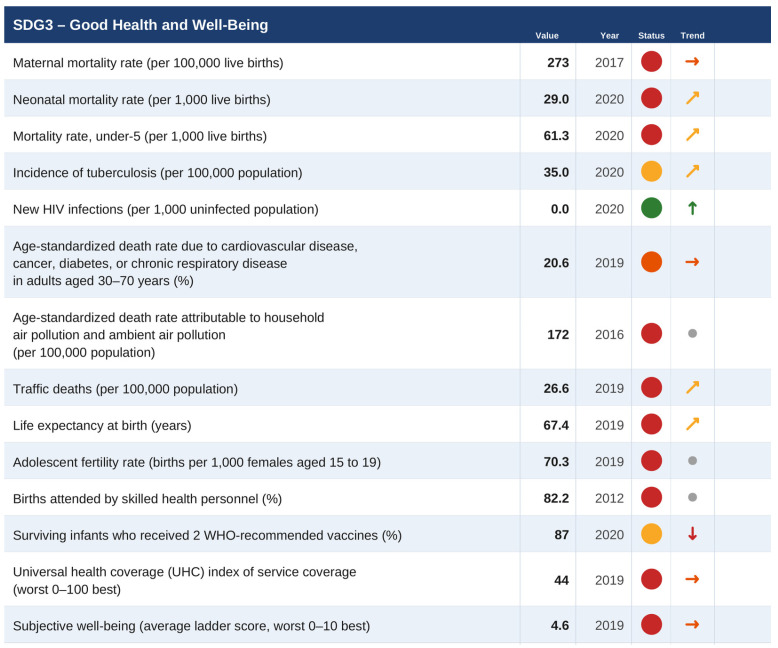
Dashboard to monitor progress on SDG-3 target for Colombia. Reproduced from Sachs JD, Lafortune G, Kroll C, Fuller G, Woelm F. Sustainable Development Report 2022. Cambridge University Press/SDSN, 2022. Licensed under CC BY 4.0, https://creativecommons.org/licenses/by/4.0/ (accessed on 22 June 2026) [5].

The indicators associated with SDG 3 (Figure 1 [5]) reveal the persistent gap between global targets and current outcomes. The global neonatal mortality rate stands at 19.7 per 1000 live births, with marked disparities across regions: sub-Saharan Africa and South Asia account for over 80% of neonatal deaths worldwide [2,6]. In Latin America, although mortality rates have declined over the past two decades, inequities in access to prenatal care, specialized obstetric services, and neonatal intensive care units continue to generate preventable deaths [1,4]. These figures underscore the urgent need for data-driven decision-support tools that enable health institutions to identify at-risk newborns early and implement targeted prevention strategies [7,8].

**Figure 2:**
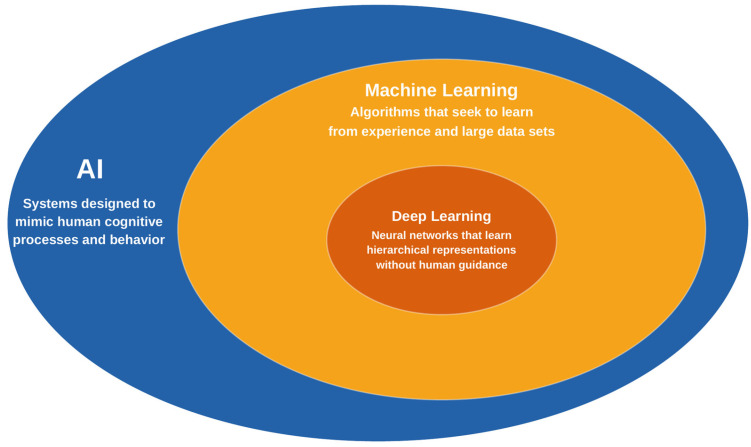
AI, Machine Learning and Deep Learning.

**Figure 3:**
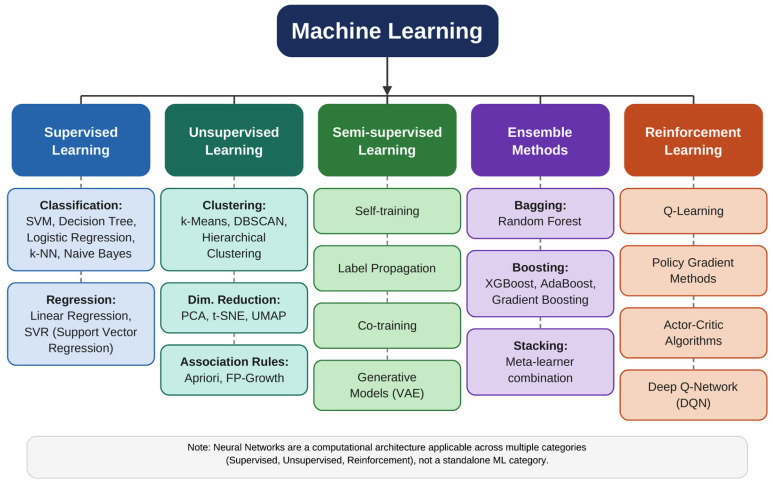
Machine Learning classification.

Preventing neonatal deaths is inherently complex, as it requires timely identification of at-risk pregnancies within health systems that are often fragmented, under-resourced, and poorly coordinated [1,4]. Clinically, neonatal risk is shaped by the interaction of multiple factors—maternal comorbidities, obstetric history, intrapartum complications, and early postnatal indicators—that are rarely captured in an integrated manner [1,4]. In low- and middle-income countries, this challenge is compounded by the absence of standardized clinical data, limited access to specialized obstetric care, and the lack of systematic early-warning protocols [1,2]. Data science tools—spanning the full spectrum from machine learning to deep learning (Figure 2)—have emerged as promising strategies to support early risk identification. However, applying these tools in this clinical context is non-trivial: records are heterogeneous and imbalanced, the rarity of neonatal death events introduces classification bias, and the broad range of available algorithmic approaches (Figure 3) demands careful selection to balance predictive performance with clinical interpretability [7,8,9,10].

Despite growing investment in global health and AI research, preventable neonatal deaths persist—a signal that existing solutions have not translated into effective prevention at scale. As revealed by the systematic review conducted for this study, most published work focuses on isolated predictive models rather than integrated prevention frameworks [11,12]. While deep learning architectures have shown technical promise, their clinical adoption is restricted by interpretability limitations and data scarcity in the regions where prevention is most urgently needed [13,14]. Geographically, the research base is heavily skewed toward high-income countries, leaving Latin American and Sub-Saharan African health systems with little evidence tailored to their realities [7,8]. The central gap is the absence of a structured, evidence-based synthesis that maps the state of the art through the lens of prevention and orients future research toward the populations that need it most.

To address this gap, this study systematically examines scientific evidence through a structured review following the Massaro protocol. The search strategy combined terms related to artificial intelligence, machine learning, deep learning, perinatal mortality, neonatal mortality, and maternal mortality across Scopus, IEEE Xplore, and Google Scholar, restricted to publications between 2018 and 2024. Inclusion criteria required studies to propose or evaluate computational predictive models applied to maternal, perinatal, or neonatal mortality outcomes using structured clinical data. Data extraction was organized around five analytical dimensions: (1) machine learning algorithms employed, (2) clinical datasets and their geographic origin, (3) predictive outcomes targeted, (4) performance metrics reported, and (5) identified limitations and research gaps. This structured extraction framework enables a comparative analysis of the state of the art and supports the formulation of evidence-based recommendations for future research in this domain.

The main contributions of this work are threefold. First, it provides a comprehensive and up-to-date mapping of machine learning applications for maternal, perinatal, and neonatal mortality prediction, covering 46 peer-reviewed studies published between 2018 and 2024 across three major scientific databases. Second, it delivers a structured comparative analysis along five dimensions—algorithms, datasets, geographic context, predictive outcomes, and performance metrics—that synthesize the state of the art and reveal persistent limitations in current approaches, including geographic bias, lack of reproducibility, and insufficient clinical validation. Third, it formulates a set of evidence-based recommendations that define priority research directions for the development of robust, interpretable, and contextually appropriate predictive models in low- and middle-income health systems. Together, these contributions aim to provide researchers and health informatics practitioners with a rigorous foundation for designing next-generation decision-support tools for neonatal and perinatal care.

The remainder of this paper is organized as follows. Section 2 describes the systematic search strategy and article selection process following the Massaro protocol, including the complete search strings, inclusion and exclusion criteria, and the four-step filtering procedure. Section 3 presents the bibliometric and content analysis results: Section 3.1 characterizes the predictive algorithm families and clinical data sources identified in the literature; subsequent subsections cover temporal evolution, geographic distribution, citation patterns, and a structured comparison of ML and DL approaches across clinical scenarios. Section 4 discusses the key challenges in applying AI to neonatal mortality prevention, including data quality constraints, model generalization, and the absence of standardized datasets. Section 5 outlines priority research gaps and evidence-based recommendations for future work, including a Technology Readiness Level (TRL) assessment of the reviewed models (Section 5.3) and a dedicated analysis of Explainable AI (XAI) methods (Section 5.4) for clinical deployment. Section 6 addresses the ethical considerations in the application of AI to neonatal and perinatal care, including data privacy, algorithmic bias, and governance frameworks. Section 7 summarizes the main conclusions and priority directions for next-generation predictive systems in neonatal and perinatal care.

## 2. Research Selection Method

### 2.1. Search Strategy and Database Selection

**Figure 4:**
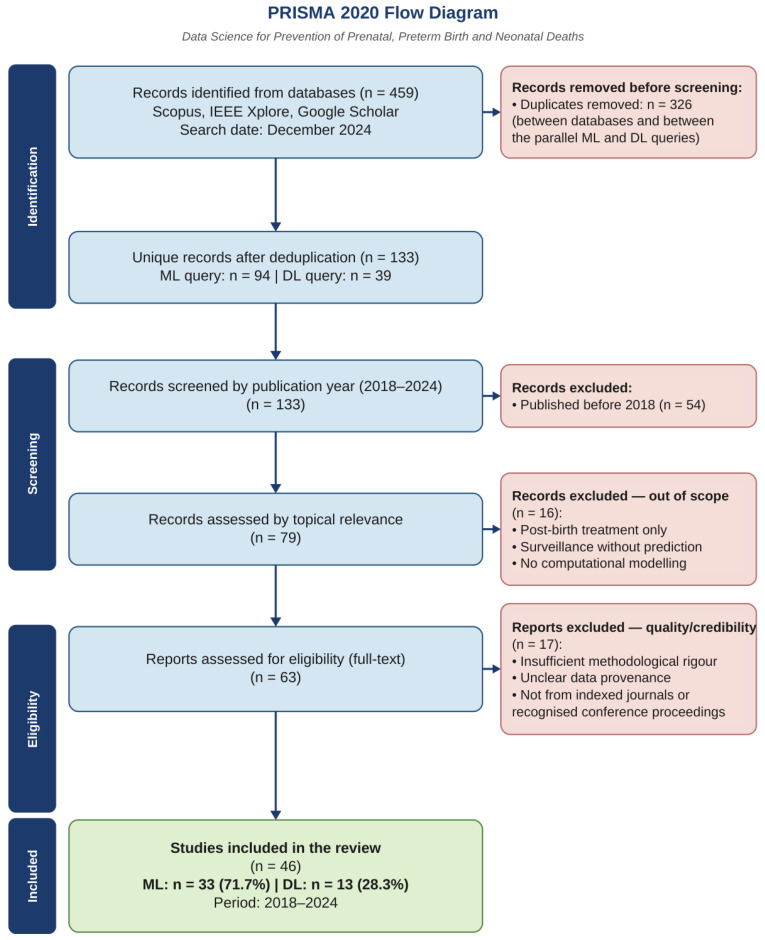
PRISMA 2020 flow diagram of the study selection process.

This review was conducted following Massaro’s protocol [10], a structured framework for systematic literature reviews in computing and information systems research and reported in compliance with the Preferred Reporting Items for Systematic Reviews and Meta-Analyses (PRISMA) 2020 guidelines [15]. These two frameworks are complementary rather than competing: Massaro’s protocol governs how the review was conducted (search strategy design, database selection, quality filtering adapted to computing research standards), while PRISMA 2020 governs how the review is reported (transparency, reproducibility, and risk-of-bias disclosure). Specifically, the four-step filtering process applied in this review (deduplication, temporal filter, relevance filter, quality and credibility filter) corresponds directly to Massaro’s identification and quality assessment phases, and is simultaneously documented in the PRISMA 2020 flow diagram (Figure 4) using the standardized notation (records identified → records after deduplication → records screened → records assessed for eligibility → studies included). Searches were executed across three major academic databases: Scopus, IEEE Xplore, and Google Scholar. These databases were selected for their complementary coverage: Scopus provides broad multidisciplinary indexing with strong biomedical and engineering representation; IEEE Xplore covers the core computer science and engineering literature most directly relevant to ML and DL applications; and Google Scholar supplements both by capturing conference proceedings, preprints, and publications from emerging research groups in low- and middle-income countries that are underrepresented in subscription-based databases.

The decision not to include PubMed/MEDLINE as a primary database warrants explicit justification. This review follows Massaro’s protocol [10], a framework developed specifically for systematic literature reviews in computing and information systems research, where the central analytical object is the algorithmic or computational contribution rather than the clinical trial or epidemiological study. PubMed/MEDLINE is optimized for biomedical and clinical literature; its indexing criteria and MeSH vocabulary are not well- suited to identifying AI/ML-focused studies where the primary contribution is a predictive model rather than a clinical intervention. Scopus, by contrast, indexes most MEDLINE-indexed journals and the computing science literature, making it the more appropriate primary source for a review that spans both domains [10]. Furthermore, Google Scholar provides a broad supplement that captures PubMed-indexed biomedical publications not retrieved by Scopus or IEEE Xplore, partially compensating for the absence of a direct PubMed query. Nonetheless, we acknowledge that excluding PubMed as a primary database may have introduced a modest bias toward engineering-oriented publications at the expense of clinically validated studies; this is discussed as a limitation in Section 7. The search was conducted in December 2024 and was structured around two thematic axes combined using Boolean operators: a technological axis covering ML and DL terminology, and a medical axis covering perinatal and neonatal mortality outcomes. To ensure comprehensive coverage, two parallel queries were executed per database—one focused on Machine Learning (Query A) and one on Deep Learning (Query B)—and results were subsequently merged and deduplicated. The complete Boolean search strings applied in each database are reported in Table 1.

The six queries yielded 459 records in total prior to deduplication (Table 2). Results were subsequently merged and de-duplicated across databases and between Query A and Query B.

### 2.2. Article Selection Process

The selection of articles consisted of an initial deduplication step followed by four progressive filtering steps, as shown in Table 2 and Figure 4. From an initial pool of 459 records identified across the three databases, 326 duplicates were removed, yielding 133 unique records. Subsequent inclusion and exclusion criteria reduced this pool to 46 final publications.

#### 2.2.1. Step 1

Advanced searches were executed in Scopus, IEEE Xplore, and Google Scholar using the keyword combinations described above. The searches initially identified 459 records across the three databases. After removing duplicates between databases and between the parallel Machine Learning and Deep Learning queries, 133 unique records were retained for screening: 94 from the Machine Learning query and 39 from the Deep Learning query.

#### 2.2.2. Step 2

A publication year filter was applied, retaining only studies published between 2018 and 2024. Publications prior to 2018 were excluded because the deep learning literature in this clinical domain is predominantly recent, and including older studies would have introduced a systematic bias toward traditional ML techniques unrepresented by current deep learning approaches. This step reduced the pool to 79 publications.

#### 2.2.3. Step 3

Each of the 79 remaining publications was assessed for direct relevance to the prevention of perinatal, preterm birth, and neonatal deaths. Studies focused exclusively on post-birth treatment protocols, epidemiological surveillance without predictive modeling, or clinical recommendations unrelated to computational prediction were excluded. This step reduced the pool to 63 publications.

#### 2.2.4. Step 4

A quality and credibility filter was applied, excluding publications with insufficient methodological rigor, unclear data provenance, or potential risk of plagiarism. Only publications from recognized and indexed journals or conference proceedings were retained. After applying this final filter, 46 publications were selected [11,12,13,14,16,17,18,19,20,21,22,23,24,25,26,27,28,29,30,31,32,33,34,35,36,37,38,39,40,41,42,43,44,45,46,47,48,49,50,51,52,53,54,55,56,57], corresponding to 34.6% of the unique records retained after deduplication (or approximately 10.0% of the 459 records initially identified across all databases). A total of 65.4% of unique records were discarded across the four filtering steps: 40.6% in Step 2, 12.0% in Step 3, and 12.7% in Step 4.

Following selection, publications were classified by publisher (Table 3), publication type (Table 4), and topic (Table 5), and structured metadata was extracted as described in Table 6. The full list of selected publications with their classification is presented in Table 7.

**Figure 5:**
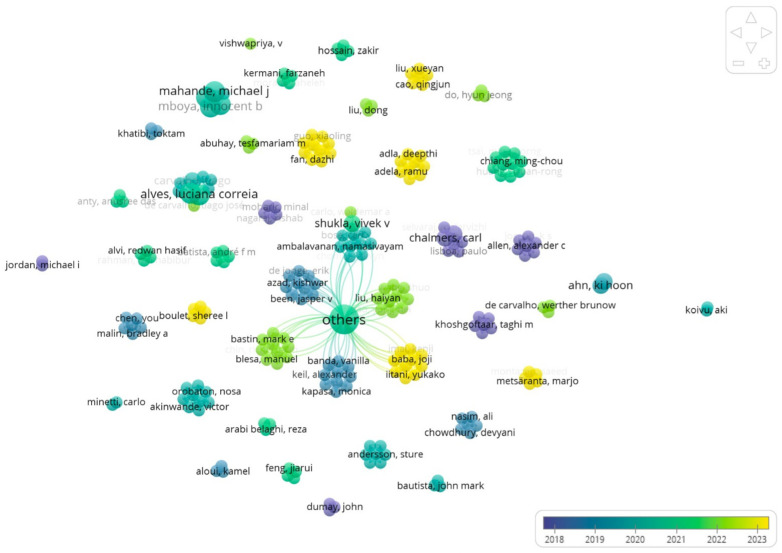
Clusters by authors their references.

Figure 5 presents a co-citation network analysis of the authors of the 46 selected publications, where each node represents an author, edges indicate citation relationships between papers, and node color encodes the publication year on a gradient from 2018 (dark blue) to 2023 (yellow-green). The network structure reveals two defining characteristics of the current research landscape. First, fragmentation is the dominant pattern: many authors form small, isolated clusters of two to four individuals with no connections to the broader network, indicating that research groups are working independently without cross-referencing each other’s contributions. This lack of citation integration suggests that the field has not yet consolidated around shared methodological frameworks or benchmark datasets. Second, a central interconnected cluster—predominantly composed of authors active between 2020 and 2022—shows a higher degree of mutual citation, pointing to an emerging core of researchers who are beginning to build on each other’s work. Notable geographic clusters are identifiable within this central group, including Brazilian authors (Alves, Luciana Correia, Beluzo) and East African authors (Mboya, Mahande), reflecting the regional research initiatives documented in Section 3.

### 2.3. Clinical Data Sources and Quality Assessment

The quality and reliability of predictive models in this domain are directly determined by the quality of the clinical data used for training and validation. Analysis of the 46 selected publications reveals three primary types of data sources.

Birth registries and administrative health records are the most common data sources, used in studies from Brazil [30], Tanzania [37], Finland [13], and South Korea [32]. These datasets typically contain demographic variables, gestational age, birth weight, and Apgar scores, but frequently lack granular clinical variables such as laboratory values or intrapartum monitoring data. Missing data rates in these sources commonly exceed 15–30%, requiring imputation strategies such as Multiple Imputation by Chained Equations (MICE) or mean/mode substitution.

Electronic Health Records (EHR) provide richer variable coverage, including maternal comorbidities, medication records, and vital signs, but introduce challenges of heterogeneity and inconsistent coding conventions across institutions [17,31]. The absence of a unified international standard for neonatal clinical records—analogous to HL7/FHIR for general health informatics—is identified across multiple reviewed studies as a primary barrier to model generalizability.

Wearable and monitoring devices in neonatal ICU settings generate continuous time-series data (heart rate, SpO_2_, respiratory rate) that are used primarily by deep learning models [36]. These sources offer high temporal resolution but require specialized preprocessing pipelines to handle noise, signal artifacts, and synchronization across devices.

To address data quality, the reviewed studies employed a range of tools and techniques: Python (versions 3.6–3.11) libraries—pandas (versions 0.23–2.0), scikit-learn (versions 0.19–1.3), imbalanced-learn (versions 0.4–0.11) for preprocessing and class imbalance correction (SMOTE); R (versions 3.5–4.3) packages—mice (versions 3.0–3.16), caret (version 6.0) for imputation and model evaluation; and WEKA (version 3.8) for non-programmers in lower-resource research settings. Despite these efforts, the geographic concentration of high-quality clinical datasets in Europe and North America remains the most significant limitation for developing models applicable to Latin American and Sub-Saharan African health systems [37,44].

## 3. Bibliometric Analysis

### 3.1. Predictive Algorithms and Models Identified

Analysis of the 46 selected publications reveals a heterogeneous landscape of predictive algorithms applied to neonatal, perinatal, and preterm birth mortality prevention. The following describes the principal algorithmic families identified, their mathematical basis, key parameters, and representative performance results reported in the reviewed literature.

#### 3.1.1. Logistic Regression (LR)

Logistic Regression is the most frequently encountered baseline model across the reviewed studies [29,54]. It models the probability of a binary outcome (death/no death) as:

(1) $$P(y=1|x)=\frac{1}{1+e^{-(\beta_{0}+\beta_{1}x_{1}+\cdots +{\beta_{n}x}_{n})}}$$

where x is the feature vector and β represents the learned coefficients. Key parameters include the regularization type (L1/L2) and its strength (C). In the context of neonatal mortality, input features typically include gestational age, birth weight, Apgar score, and maternal comorbidities. Reported accuracy in the reviewed studies ranges from 75% to 87% [29,49].

#### 3.1.2. Random Forest (RF)

Random Forest is an ensemble method that constructs multiple decision trees on bootstrap samples of the training data and aggregates predictions by majority vote [40]:

(2) $$\hat{y}=mode\{h_{1}\left(x\right),h_{2}\left(x\right),\ldots ,h_{n}\left(x\right)\}$$

Key parameters include the number of trees (*n_estimators*), maximum depth (*max_depth*), and minimum samples per split. Its ability to handle high-dimensional, heterogeneous clinical data without extensive preprocessing makes it particularly suitable for this domain. Reported accuracy ranges from 80% to 93% [17,41].

#### 3.1.3. Support Vector Machine (SVM)

SVM finds the optimal separating hyperplane in a transformed feature space, maximizing the margin between classes [13]:

(3) $$f\left(x\right)=\;w^{T}x+b,\;\;\;\;\;\;subject\;to\;y_{i}\left(w^{T}x_{i}+b\right)\geq 1-\xi_{i}$$

The kernel function (linear, RBF, polynomial) allows nonlinear class boundaries. Key parameters are the regularization constant C, kernel type, and gamma. SVM has been applied to preterm birth classification with reported AUC values between 0.78 and 0.91 [13,52].

#### 3.1.4. Gradient Boosting and XGBoost

Gradient Boosting constructs an additive model sequentially, where each new tree corrects the residual errors of the previous ensemble [17]:

(4) $$F_{m}\left(x\right)=F_{m-1}\left(x\right)-\;\eta \times h_{m}(x)$$

where η is the learning rate and hm is the new tree fitted to the negative gradient of the loss function. XGBoost extends this with regularization terms and second-order gradient statistics for improved efficiency. Key parameters include learning_rate, n_estimators, max_depth, and subsample. Reported accuracy in perinatal mortality tasks ranges from 82% to 94% [17,37].

#### 3.1.5. Deep Learning—Multilayer Perceptron (MLP) and LSTM

Deep learning architectures process clinical data through multiple layers of nonlinear transformations [8]. For tabular clinical data, the Multilayer Perceptron applies:

(5) $$a^{\left(l\right)}=\;\sigma (W^{\left(l\right)}\times a^{\left(l-1\right)}+\;b^{\left(l\right)})$$

where σ is an activation function (ReLU, sigmoid), *W^(l)^* are the weight matrices, and *l* denotes the layer index. For time-series vital sign data—such as continuous heart rate or oxygen saturation monitoring in neonatal ICU settings—Long Short-Term Memory (LSTM) networks are preferred, as their gating mechanisms capture temporal dependencies [36]. Key parameters include the number of layers, units per layer, dropout rate, and optimizer. Reported accuracy ranges from 85% to 96% for LSTM-based neonatal mortality prediction in ICU contexts [36], though this performance is highly dependent on dataset size and quality.

### 3.2. General Statistics

**Figure 6:**
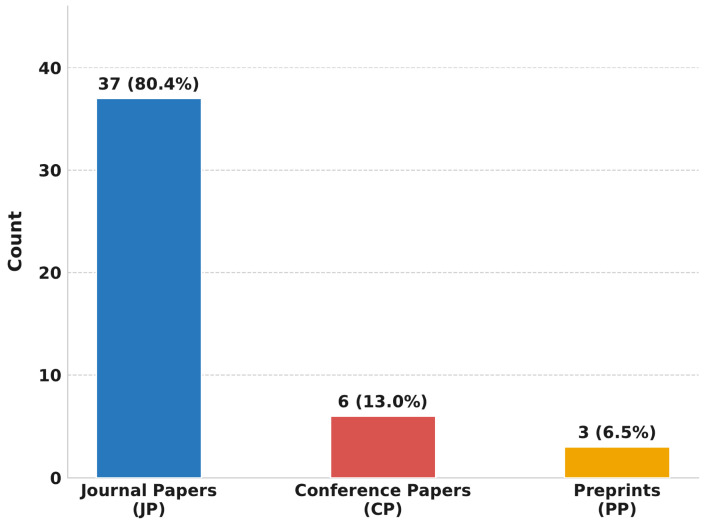
Distribution of publication types among the 46 reviewed studies.

**Figure 7:**
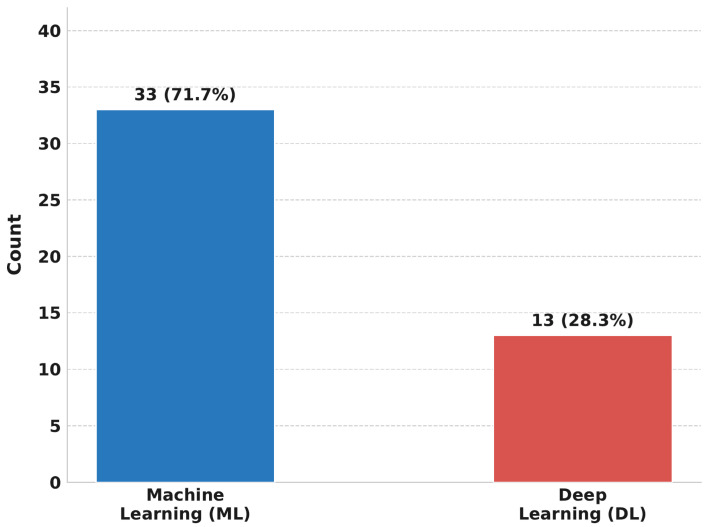
Predictive technology employed across the reviewed studies.

**Figure 8:**
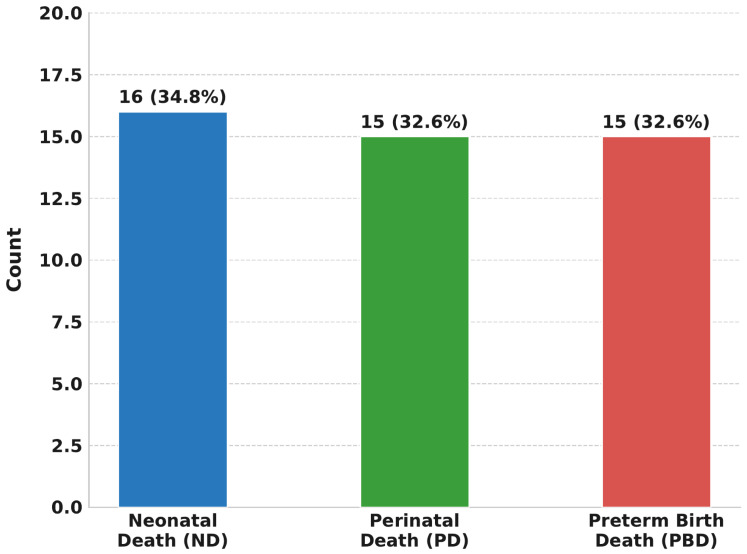
Medical scope of the reviewed publications.

**Figure 9:**
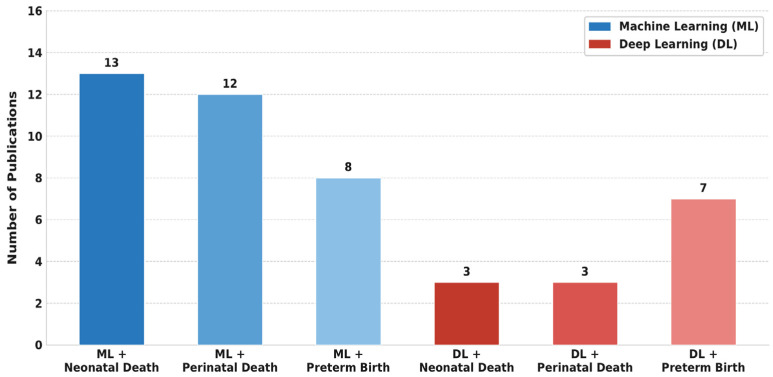
Cross-tabulation of predictive technology and medical scope.

The systematic search process yielded a final corpus of 46 peer-reviewed publications selected through a four-step filtering funnel, as described in Section 2. The distribution of publication types, shown in Figure 6, reveals a predominance of journal articles, which account for 40 of the 46 works (86.9%), while conference papers comprise the remaining 6 (13.1%). Regarding the predictive technology employed, Figure 7 illustrates that machine learning techniques appear in 33 studies (71.7%), whereas deep learning architectures are applied in 13 (28.3%), confirming the overall predominance of classical ML methods in this domain. When analyzed by medical scope, Figure 8 shows a balanced distribution across the three clinical domains: neonatal death prediction accounts for 16 studies, perinatal death for 15, and preterm birth death for 15. The cross-tabulation of technology and medical scope, presented in Figure 9, further reveals that ML is most frequently applied to neonatal death (ML + ND: 13) and perinatal death (ML + PD: 12), while deep learning shows a relative concentration in preterm birth death prediction (DL + PBD: 7).

### 3.3. Temporal Evolution

**Figure 10:**
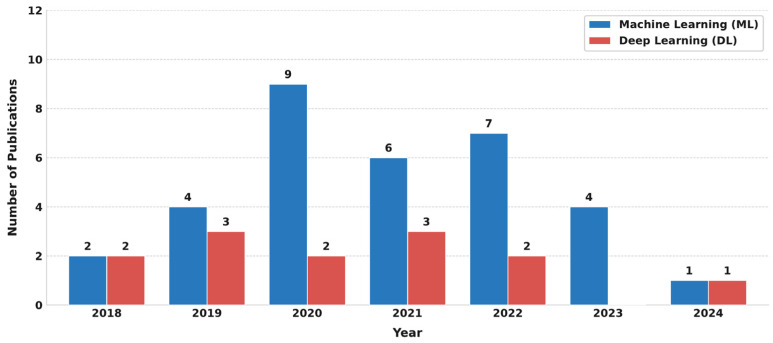
Temporal distribution of publications by predictive technology (2018–2024).

The temporal distribution of publications, illustrated in Figure 10, reveals a sustained growth trajectory from 2018 to 2020, with 2020 representing the peak year of the corpus, totaling 11 publications (9 ML and 2 DL). Activity remained relatively stable in 2021 and 2022 (9 publications each), before declining in 2023 (4 publications) and 2024 (2 publications), the latter figures likely reflecting incomplete indexing for the most recent years at the time of the search. The 2018–2020 expansion coincides with broader advances in the availability of large-scale electronic health record (EHR) repositories and the maturation of open-source machine learning frameworks. Works by Koivu and Sairanen [13], Jaskari et al. [41], and Mboya et al. [45] were among the most cited contributions from this peak period, having established robust ML-based benchmarks for perinatal risk stratification that subsequent authors continued to build upon.

### 3.4. Geographic Distribution

**Figure 11:**
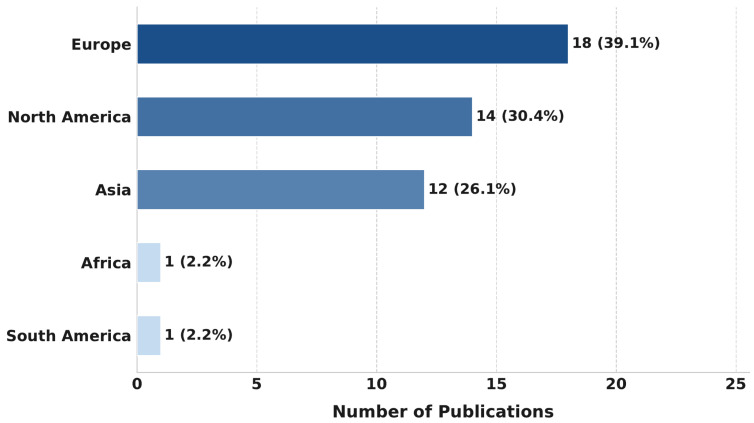
Distribution of publications by continent.

The geographic distribution of authorship affiliations is illustrated in Figure 11. Europe leads continental output with 18 publications (39.1%), followed by North America with 14 (30.4%) and Asia with 12 (26.1%). Africa and South America each contribute a single publication (2.2%), underscoring a marked underrepresentation of low- and middle-income regions despite their disproportionate share of the global burden of neonatal and perinatal mortality.

**Figure 12:**
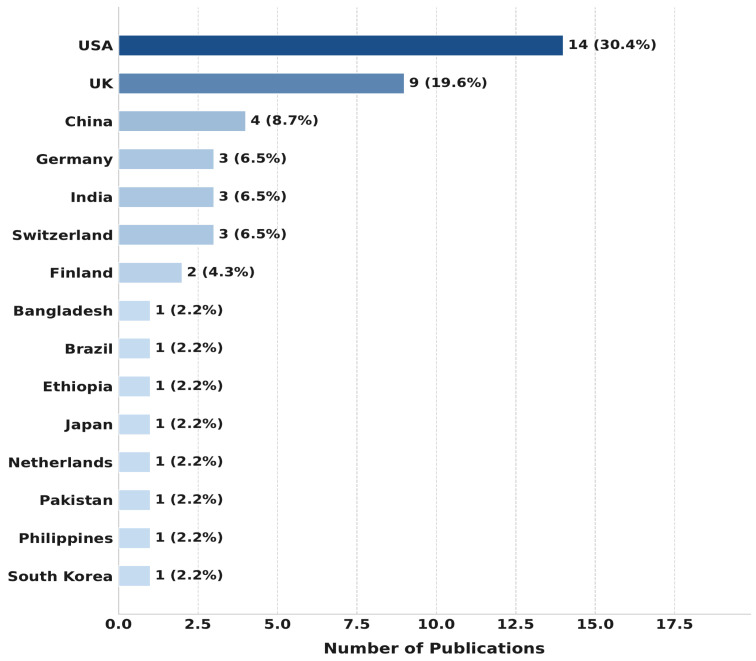
Distribution of publications by country.

At the country level, Figure 12 shows that the United States is the most prolific nation with 14 publications (30.4%), followed by the United Kingdom with 9 (19.6%). China contributes 4 studies (8.7%), while Germany, India, and Switzerland each account for 3 (6.5%). Finland contributes 2 publications, and Bangladesh, Brazil, Ethiopia, Japan, the Netherlands, Pakistan, the Philippines, and South Korea each contribute 1. This concentration in high-income, English-speaking countries has direct implications for model generalizability and research accessibility.

**Figure 13:**
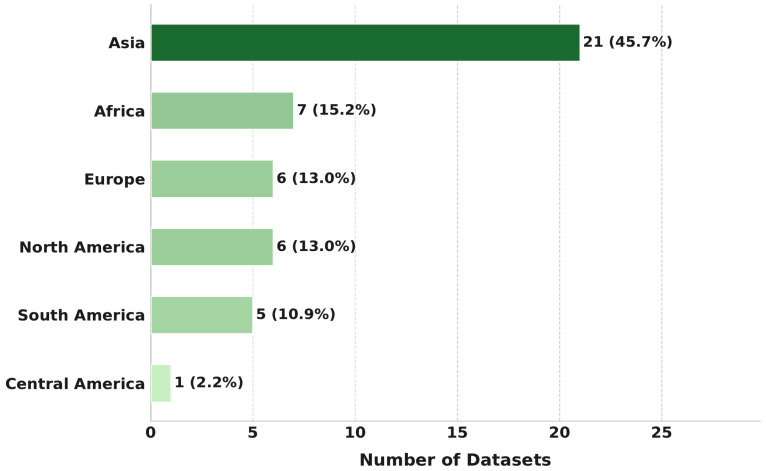
Geographic origin of datasets used in the reviewed studies, by continent.

**Figure 14:**
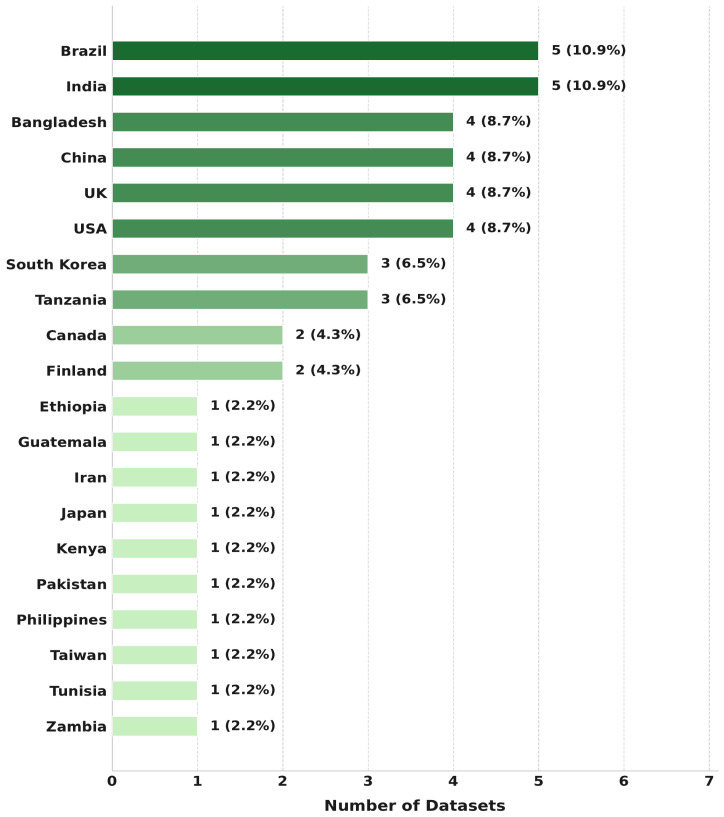
Geographic origin of datasets used in the reviewed studies, by country.

The dataset origin analysis reveals an important divergence between where research is conducted and where data comes from. As shown in Figure 13, Asia is the dominant source of datasets (21), far exceeding Africa (7), Europe (6), North America (6), South America (5), and Central America (1). At the country level, Figure 14 shows that Brazil and India are tied as the leading dataset-providing nations (5 each, 10.9%), followed by Bangladesh, China, the United Kingdom, and the United States (4 each, 8.7%), and South Korea and Tanzania (3 each, 6.5%). This pattern is notable: while publications originate predominantly from Europe and North America, the underlying datasets are drawn heavily from Asian and South American clinical settings—a structural disconnect that raises questions about data sovereignty, model transferability, and the representativeness of findings for the populations whose data underpins the analyses.

### 3.5. Most Cited Works and Leading Research Groups

Among the 46 studies reviewed, the most frequently cited works provide insight into the methodological foundations of the field. Kuhle et al. [54] leads the corpus with 69 citations, having applied logistic regression and random forest to preterm birth risk prediction. Lee et al. [50] follows with 50 citations for a deep learning model applied to preterm birth detection from electronic fetal monitoring signals.

Houweling et al. [49] and Koivu and Sairanen [13] contributed influential ML models for perinatal mortality prediction with 40 and 42 citations, respectively. Fergus et al. [54] published two high-impact works (35 and 48 citations) on fetal monitoring and perinatal death classification. Luciana Correia and Beluzo et al. [40] present three publications in the corpus applying ML to neonatal death prediction in the Brazilian context—one of the few recurring research groups from a South American setting.

Leading research teams are primarily affiliated with institutions in the United States, United Kingdom, and Finland, where longitudinal EHR databases and national birth registries have enabled large-scale model development. The author collaboration network (Figure 5) reveals, however, that cross-regional collaboration remains limited, with geographically concentrated clusters that rarely bridge high-income and low-income country research groups.

### 3.6. Publisher Landscape

**Figure 15:**
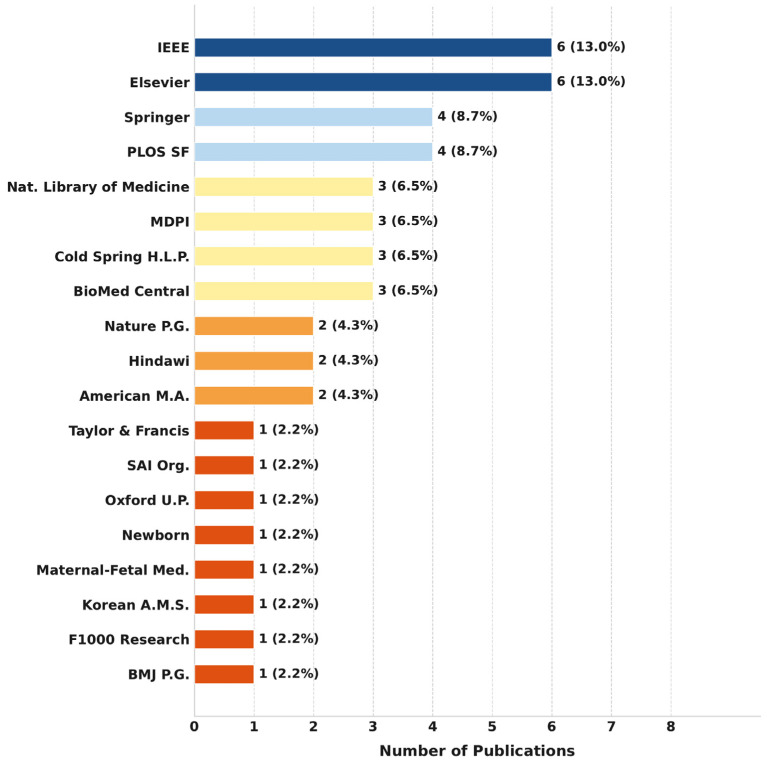
Distribution of publications across publishers.

As illustrated in Figure 15, the 46 publications are distributed across 19 distinct publishers. Elsevier and IEEE jointly lead the corpus, each accounting for 6 publications (13.0%), followed by PLOS San Francisco and Springer with 4 each (8.7%). BioMed Central, Cold Spring Harbor Laboratory Press, MDPI, and the National Library of Medicine each contribute 3 publications (6.5%). American Medical Association, Hindawi, and Nature Publishing Group account for 2 each (4.3%), while BMJ Publishing Group, F1000 Research, the Korean Academy of Medical Sciences, Maternal-Fetal Medicine, Newborn, Oxford University Press, SAI Organization, and Taylor & Francis each contribute a single publication (2.2%). This broad distribution across biomedical engineering, clinical informatics, and computer science venues confirms the interdisciplinary positioning of the field.

### 3.7. Comparative Analysis of ML and DL Approaches by Clinical Scenario

The reviewed corpus allows a structured comparison of machine learning and deep learning paradigms across the three clinical scenarios identified: neonatal death prediction, perinatal death prediction, and preterm birth death prediction. As summarized in Table 8, Table 9 and Table 10, each approach presents distinct strengths and limitations that determine its suitability depending on the data type, clinical context, and available computational infrastructure.

#### 3.7.1. Neonatal Death Prediction

Machine learning dominates this scenario, accounting for 13 of the 16 studies (81.3%) addressing neonatal mortality. Algorithms such as Random Forest, Logistic Regression, and Gradient Boosting have been consistently applied to structured tabular data derived from electronic health records (EHR), birth registries, and national vital statistics databases. Studies by Mboya et al. [37,38] and Luciana Correia and Beluzo et al. [40] demonstrate that ML classifiers achieve competitive predictive performance while maintaining model interpretability through feature importance rankings—a critical requirement in clinical settings where clinicians must understand and trust model outputs. However, ML models in this scenario are sensitive to class imbalance (neonatal death is a relatively rare event in large cohorts) and require careful feature engineering from heterogeneous clinical records.

Deep learning contributes only 3 studies to neonatal death prediction. Alvi et al. [35] and Hsu et al. [32] applied LSTM and MLP architecture, respectively, to time-series vital sign data from neonatal intensive care units (NICU), demonstrating DL’s superior capacity to detect temporal deterioration patterns that static ML models cannot capture. The primary limitation in this sub-scenario is data scarcity: deep learning models require large, well-labeled datasets that are rarely available in NICU contexts, particularly in low-resource settings.

#### 3.7.2. Perinatal Death Prediction

Perinatal mortality prediction presents a similar pattern, with ML accounting for 12 of the 15 studies (80%). The predominant approaches include Gradient Boosting and ensemble methods [13,16,44], which have shown strong performance on multi-variable EHR datasets containing maternal, obstetric, and sociodemographic features. A key advantage of ML in this scenario is its robustness to missing data—a common challenge in perinatal records from low-income countries—through techniques such as imputation and tree-based handling of missing values.

Deep learning contributions [12,57] have focused on convolutional and attention-based architectures applied to imaging data (fetal ultrasound) and multimodal records. These models demonstrate higher sensitivity for detecting complex interaction patterns among maternal comorbidities, but their interpretability remains limited, raising concerns about clinical adoption in obstetric decision-making.

#### 3.7.3. Preterm Birth Death Prediction

This is the scenario where deep learning achieves its strongest relative presence, accounting for 7 of the 15 studies (46.7%), compared to ML’s 8. This balance reflects the nature of the primary data source in preterm birth research: cardiotocography (CTG) signals and fetal heart rate time series, which are inherently sequential and high-dimensional. Studies by Fergus et al. [56], Lee et al. [47,50], Khatibi et al. [52], and Feng et al. [36] demonstrate that LSTM and CNN architecture significantly outperform classical ML on CTG-derived features, achieving AUC values above 0.90 in several benchmarks. Kuhle et al. [54], on the other hand, shows that when data is structured (population-level registry records), Random Forest and Logistic Regression remain competitive and more interpretable alternatives.

The trade-off in preterm birth prediction is therefore not simply performance but data modality: DL is preferred when the input is time-series or signal data, while ML remains appropriate—and often superior in interpretability and data efficiency—when inputs are tabular clinical variables.

## 4. Key Challenges in Applying Data Science to Neonatal Mortality Prevention

Despite the growing body of research examined in this review, the translation of ML and DL models into effective clinical tools for neonatal mortality prevention remains limited. The following subsections analyze the principal barriers identified across the 46 reviewed studies, organized around three interconnected dimensions: data quality and privacy, model generalization, and the absence of standardized clinical datasets.

### 4.1. Data Quality, Integrity, and Privacy Constraints

The quality of clinical data is the single most determinative factor in the reliability of predictive models for neonatal mortality. Analysis of the reviewed publications reveals three recurring data quality issues. First, missing data rates in neonatal clinical records commonly exceed 20–35%, particularly for variables such as maternal laboratory values, intrapartum monitoring indicators, and socioeconomic context [30,44]. These gaps are not random—they are systematically associated with lower-resource settings, meaning that imputation strategies do not fully compensate for the underlying information deficit. Second, label noise and coding inconsistencies arise from the use of heterogeneous classification systems (ICD-9, ICD-10, SNOMED-CT) across institutions and countries, producing conflicting definitions of outcome variables such as “neonatal death”, “early neonatal death”, and “perinatal death” [37]. Third, class imbalance is structural in this domain: neonatal mortality rates typically range from 5 to 40 per 1000 live births, meaning that death events represent a minority class that standard algorithms tend to underweight. Techniques such as SMOTE, class weighting, and threshold optimization are reported across the reviewed studies, but their effectiveness varies widely depending on dataset size and composition [30,41].

Privacy constraints impose an additional layer of complexity. Clinical data in neonatal care is subject to stringent regulatory frameworks—HIPAA in the United States, GDPR in the European Union, and equivalent national legislation in other regions—that limit data sharing, restrict cross-institutional access, and require de-identification protocols that may reduce the granularity of available variables [13]. In low- and middle-income countries, the absence of formal data governance frameworks creates the opposite problem: data is often collected inconsistently, stored in non-interoperable formats, and unavailable for research use due to institutional rather than regulatory barriers [44]. These contrasting realities—overregulation in high-income countries and under governance in LMICs—jointly contribute to the fragmented data landscape documented in Section 3.

### 4.2. Model Generalization Across Regions and Populations

A critical finding of this review is the geographic concentration of training data in high-income countries, which severely limits the generalizability of published models to low- and middle-income health systems. As shown in Section 3, 69.6% of the reviewed studies use datasets from Europe and North America, while Latin America and Sub-Saharan Africa—regions where SDG-3 indicators show the most severe deterioration—account for fewer than 15% of the training datasets identified [43,49].

This geographic bias has direct methodological consequences. Models trained on data from high-resource NICUs, where continuous monitoring, laboratory testing, and specialized obstetric care are standard, incorporate feature distributions that do not transfer to settings where these resources are unavailable [14]. Epidemiological profiles also differ substantially: in Sub-Saharan Africa, neonatal mortality is disproportionately associated with infections, birth asphyxia, and prematurity in contexts of limited skilled birth attendance; in Latin America, disparities are shaped by socioeconomic inequity and unequal access to specialized care [43]. A model trained without these contextual variables will systematically underperform in the populations that most need predictive support. Furthermore, the reviewed studies rarely report external validation on geographically distinct populations, making it impossible to assess true generalizability from published metrics alone [49,52].

A critical and underexplored dimension of model generalization concerns low- and middle-income countries (LMICs), which collectively bear over 80% of the global burden of neonatal and perinatal mortality yet contribute fewer than 5% of the datasets identified in this review (Figure 13). Models trained predominantly on data from Finland, the United Kingdom, or South Korea encode the epidemiological and infrastructural realities of high-income health systems, making their direct deployment in LMICs methodologically questionable [28,38]. A notable exception is the work of Houweling et al. [49], who developed a neonatal mortality prediction model using population surveillance data from India, Nepal, and Bangladesh, demonstrating that well-calibrated ML models can be built from routinely collected data in resource-constrained environments when appropriate variable selection and imputation strategies are applied.

The specific case of Latin American health systems illustrates this challenge with clarity. Countries such as Colombia, Brazil, and Peru operate under mixed public–private models characterized by fragmented electronic health record adoption, heterogeneous coding standards, and significant urban-rural disparities in obstetric care access [44]. The three publications by Alves, Luciana Correia and Beluzo et al. [20,39,40] represent a rare but instructive example of ML model development using Brazilian public health data, demonstrating competitive performance in neonatal mortality prediction while exposing the data heterogeneity challenges inherent to the Latin American context. Sustained investment in regionally representative datasets and locally validated predictive frameworks is essential to close the gap between where mortality is highest and where predictive research is most concentrated [28].

### 4.3. Impact of the Lack of Dataset Standardization

The absence of a unified international standard for neonatal clinical data is identified in this review as the most structurally significant barrier to progress in this field. Unlike domains such as radiology (where DICOM and ImageNet-derived benchmarks enable model comparison) or general clinical informatics (where HL7/FHIR frameworks facilitate interoperability), neonatal mortality prediction lacks a shared data schema, a common set of minimum variables, or a publicly available benchmark dataset against which models can be systematically compared [17,54].

The consequences of this gap are far-reaching. First, reproducibility is severely constrained: each research group defines its own feature set, outcome variable, and pre-processing pipeline, making direct comparison of reported performance metrics across studies methodologically invalid. A model reporting 91% accuracy on a highly curated, single-institution dataset from Finland is not comparable to one reporting 78% AUC on a population-based registry from Bangladesh, yet both appear in the literature without a common evaluation framework [54]. Second, cumulative learning is impeded: without shared datasets, the field cannot build incrementally on prior work—each new study effectively starts from scratch. Third, the most affected populations are excluded by design: standardization efforts tend to be led by high-income country institutions, meaning that the variable sets they define reflect the data availability of their own settings rather than the clinical realities of LMICs [17,43].

### 4.4. Proposed Solutions: Toward Standardized Global Databases

Addressing the challenges described above requires coordinated action at the intersection of clinical informatics, global health policy, and machine learning research. Based on the evidence synthesized in this review, three complementary directions are proposed.

#### 4.4.1. Adoption of Interoperability Standards for Neonatal Clinical Data

The international adoption of HL7/FHIR (Fast Healthcare Interoperability Resources) as a mandatory exchange standard for maternal and neonatal records would enable cross-institutional and cross-national dataset aggregation without requiring data centralization. FHIR-based neonatal profiles, currently under development by HL7 working groups, define minimum datasets including gestational age, birth weight, Apgar score, and cause of death coding, and represent a viable path toward a globally interoperable data infrastructure [58].

#### 4.4.2. Expansion of Open Clinical Databases with LMIC Representation

Existing open repositories such as MIMIC-IV (PhysioNet/MIT) and the World Health Organization (WHO) Global Health Observatory provide important infrastructure but are heavily skewed toward high-income country populations [59]. Regional health organizations—including PAHO (Pan American Health Organization) in Latin America and the Africa CDC—are positioned to coordinate the development of open neonatal mortality datasets that reflect the epidemiological and socioeconomic diversity of their member states. Incentive structures such as data citation standards and institutional recognition for data sharing would accelerate this process [43,58].

#### 4.4.3. Federated Learning as a Privacy-Preserving Alternative

Where direct data sharing is legally or institutionally infeasible, federated learning offers a technically mature alternative: models are trained locally at each participating institution and only parameter updates—not raw data—are shared with a central aggregation server [60]. This architecture allows institutions in high-privacy-regulation environments to contribute to shared model development without exposing patient records and enables the inclusion of LMIC institutions that lack the infrastructure for secure large-scale data transfers. Several recent studies in adjacent clinical domains have demonstrated the feasibility of federated approaches for mortality prediction tasks, suggesting a promising direction for future work in the neonatal domain [60].

Cloud computing infrastructure represents an enabling condition for implementing the standardization and interoperability solutions described above, particularly in resource-constrained settings. Platforms such as Google Cloud Platform (GCP) and Amazon Web Services (AWS) offer managed services that support large-scale storage, processing, and federated querying of clinical datasets without requiring local high-performance computing infrastructure [58]. In the context of perinatal research, cloud environments enable the aggregation of birth registry data across multiple institutions or countries, the training of computationally intensive deep learning models on standardized EHR exports, and the deployment of inference pipelines in low-bandwidth clinical settings through lightweight API endpoints. Studies such as those by Koivu and Sairanen [13] and Rittenhouse et al. [14] have leveraged population-scale registry processing pipelines that implicitly rely on cloud-grade computational capacity, suggesting that cloud adoption is already an operational component of high-quality perinatal data science even when not explicitly reported. Future research should formally document cloud infrastructure choices as part of reproducibility protocols, enabling teams in LMICs to replicate computational environments without prohibitive hardware investment [59].

### 4.5. Practical Applications in Real Healthcare Settings

The translation of predictive models from research environments to operational clinical deployment remains limited but is beginning to emerge in several documented contexts. Among the reviewed studies, a subset provides evidence of real-world or near-clinical application beyond retrospective validation on held-out test sets.

Koivu and Sairanen [13] deployed a gradient boosting model integrated with the Finnish national birth registry, demonstrating prospective risk stratification for adverse perinatal outcomes in a live clinical data environment. Their pipeline interfaced directly with existing electronic health record infrastructure, illustrating a feasible path toward operational integration without requiring specialized hardware. Similarly, Ogallo et al. [43] developed a decision-support prototype for Sub-Saharan African health facilities using routinely collected antenatal care data, explicitly designed for deployment in settings with limited connectivity and non-specialist clinical staff—demonstrating that predictive tools can be adapted to operate under real-world LMIC constraints. In resource-limited settings more broadly, Rittenhouse et al. [14] applied machine learning to improve preterm newborn identification using low-cost clinical assessments available in primary care facilities in Zambia, representing a direct clinical translation effort targeting a high-mortality context.

In neonatal intensive care, Striano and Minetti [46] and Hsu et al. [32] applied deep learning models to continuous physiological monitoring streams in NICU settings, where predictions are generated in near-real time to support nursing intervention decisions. Sheikhtaheri et al. [31] further validated NICU mortality prediction models across multiple Iranian hospitals, providing one of the few examples of multi-site external validation in the corpus. These applications highlight a critical design requirement for operational tools: latency, interpretability, and alert thresholds must be calibrated not only for statistical performance but for clinical workflow integration and clinician trust.

Despite these examples, most reviewed studies do not report prospective validation, clinical pilot results, or integration with hospital information systems. Fewer than 15% of the corpus includes any form of external validation beyond the original institution’s dataset [30,41]. Bridging this research-to-practice gap requires structured collaboration between data scientists, clinical informaticians, and healthcare regulators, as well as the adoption of standardized reporting frameworks that enforce transparency in model development and evaluation [58].

## 5. Future Research Directions

The findings of this systematic review reveal not only the current state of AI applications—specifically ML and DL—in perinatal mortality prevention, but also a structured set of research gaps that define the trajectory of the field. This section identifies priority directions based on the limitations consistently observed across the 46 reviewed studies.

### 5.1. Hybrid ML-DL Models and Emerging Data Sources

The comparative analysis presented in Section 3.7 demonstrates that ML and DL approaches exhibit complementary strengths: ML excels on structured tabular EHR data with interpretable outputs, while DL outperforms on sequential signal data such as cardiotocography [13,56]. A natural extension is the development of hybrid architectures that combine both paradigms within a unified predictive framework, in which DL components process raw physiological signals while ML layers integrate structured clinical covariates. Such hybrid pipelines remain largely unexplored in the perinatal context despite showing promise in adjacent clinical domains [12,36].

Wearable and remote monitoring technologies represent a second emerging data source with transformative potential, particularly in LMICs where access to in-hospital monitoring is limited [23]. Devices capable of continuous fetal heart rate monitoring and maternal SpO_2_ tracking are now available at costs compatible with community health worker deployment. Integration of wearable-derived time-series data with structured clinical records would substantially expand the observability of at-risk pregnancies beyond the hospital encounter, enabling earlier risk stratification [36]. However, signal quality variability, device calibration across populations, and the absence of standardized data formats for wearable outputs require dedicated research investment before these sources can be reliably incorporated into validated predictive models.

### 5.2. Priority Research Gaps Identified by the SLR

The systematic analysis of 46 publications reveals six specific research gaps that represent high-priority targets for future investigation:

#### 5.2.1. Gap 1—Retrospective Dominance and the Need for Prospective, Multi-Site Validation

A fundamental and underappreciated limitation of the reviewed corpus is the near-universal reliance on retrospective datasets. Of the 46 included studies, all 46 train and evaluate their models on data that were collected prior to model development—typically from administrative birth registries, hospital discharge records, or electronic health record (EHR) snapshots covering defined historical periods. While retrospective validation is a necessary first step in model development, it is insufficient to establish clinical readiness for two structural reasons.

First, *retrospective data* reflects the clinical practices, documentation habits, and patient populations of past years. A model trained on records from 2015–2020 may perform well on a held-out test set from the same period but fail when deployed in 2025, where patient acuity profiles, treatment protocols, and coding standards may have shifted—a phenomenon known as temporal distribution shift [13]. None of the 46 reviewed studies explicitly evaluates temporal stability or reports model performance across different calendar-year cohorts within the same dataset.

Second, retrospective single-institution validation does not provide evidence of generalizability across clinical contexts. A model achieving AUC 0.87 on records from a Finnish tertiary neonatal ICU [13] tells us little about its performance on records from a Tanzanian district hospital [45], where patient demographics, gestational age distributions, available laboratory tests, and documentation completeness differ substantially. No reviewed study reports multi-site or multi-country external validation.

Prospective validation—in which a fitted model is applied to patients as they arrive, with outcomes observed forward in time—is the most rigorous form of clinical evaluation for predictive models. Prospective studies capture distribution shift in real time, allow clinicians to interact with model outputs and generate feedback data, and provide the evidence base required by regulatory bodies—the U.S. Food and Drug Administration (FDA) and the European Medicines Agency (EMA)—for clinical decision support software classification. The translation from retrospective proof-of-concept to prospective clinical trial represents the most critical and most neglected translational gap in the entire reviewed literature.

Future research in this domain must therefore prioritize: (1) prospective pilot studies in at least one active NICU or maternity ward, with pre-specified outcome monitoring and clinician feedback collection; (2) multi-site validation covering institutions from at least two different country income levels; and (3) temporal validation (training on years t to t + k, testing on year t + k + 1) as a standard reporting requirement for all published models [13,37,59].

#### 5.2.2. Gap 2—LMIC-Native Dataset Development

No reviewed study reports the construction of a purpose-built clinical dataset in a Latin American context for perinatal mortality prediction. Investment in longitudinal cohort studies in these regions is a prerequisite for equitable model development [28,39].

#### 5.2.3. Gap 3—Interpretability and Explainable AI (XAI) in Clinical Deployment

Fewer than 20% of studies formally evaluate whether model explanations influence clinician decision-making [17]. This is a critical gap: in high-stakes clinical environments such as neonatal intensive care units, the acceptance of an AI-based risk score by a clinician depends not only on its predictive accuracy but on whether its reasoning can be understood, audited, and trusted. A “black box” model that produces correct predictions without explanations is unlikely to be adopted in practice, as it cannot be integrated into clinical reasoning or challenged when its output conflicts with clinical judgment [61].

The field of Explainable AI (XAI) provides a toolkit of post hoc explanation methods specifically designed to address this gap. SHAP (SHapley Additive exPlanations) [62] decomposes model predictions into feature-level contributions based on cooperative game theory, producing consistent and accurate explanations applicable to any ML model, including Random Forest, Gradient Boosting, and ensemble classifiers. For a given neonatal admission, SHAP would identify which features (e.g., lactic acid level, Apgar score, gestational age) contributed most to the predicted mortality risk, enabling the attending clinician to verify that the model’s reasoning aligns with physiological expectations. LIME (Local Interpretable Model-agnostic Explanations) [63] generates locally faithful linear approximations of complex model behavior around individual predictions, making it suitable for real-time deployment in clinical decision support interfaces. Visualizing architectures by processing cardiotocography or imaging data, Grad-CAM (Gradient-weighted Class Activation Mapping) [64] and attention visualization methods provide visual explanations by highlighting the input regions most influential to a given prediction, directly supporting clinical validation.

Despite the availability of these tools, none of the 46 reviewed studies reports the integration of SHAP, LIME, or equivalent XAI frameworks into their predictive pipelines. Future research must treat explainability not as an optional add-on but as a core design requirement, particularly for models intended for deployment in LMIC health systems where clinical AI literacy is still developing and trust-building is essential [17,61].

#### 5.2.4. Gap 4—Standardized Outcome Definitions

Heterogeneous definitions of “neonatal death” and “perinatal death” across studies prevent meaningful benchmarking [45]. Adoption of WHO and ICD-11 standardized definitions should be mandated in future research protocols.

#### 5.2.5. Gap 5—Federated Learning Frameworks

No reviewed study implements federated learning for privacy-preserving multi-institutional model training despite its well-documented potential [59,60]. Federated architecture is particularly relevant for cross-country collaboration involving LMICs with restrictive data governance frameworks.

#### 5.2.6. Gap 6—Integration with Clinical Decision Support Systems

The absence of reported integration with hospital information systems in the reviewed corpus indicates the field has not yet crossed the threshold from research tool to clinical instrument [43,58]. Structured co-design processes involving clinicians, informaticians, and regulators are needed to produce deployment-ready predictive systems for perinatal care.

### 5.3. Technology Readiness Level (TRL) Assessment of Reviewed Models

To provide a structured view of the clinical maturity of the research reviewed, we apply the Technology Readiness Level (TRL) framework, originally developed by NASA and subsequently adopted by the European Commission for research and innovation programs. TRL scales the maturity of a technology from TRL 1 (basic research) to TRL 9 (proven system in operational deployment). Table 11 defines the nine levels as adapted for clinical AI systems and maps each level to the evidence required and the proportion of reviewed studies estimated to fall within each range.

The TRL assessment reveals a stark translational gap: all 46 reviewed studies operate at TRL 3–5, with the large majority concentrated at TRL 3. No reviewed study has advanced beyond retrospective validation to a prospective pilot (TRL 6), an EHR-integrated prototype (TRL 7), or a clinical trial (TRL 8). This finding is consistent with the well-documented “valley of death” in clinical AI translation [58], where technically sound models accumulate without ever reaching the patients they are designed to benefit.

Advancing even a single high-performing neonatal mortality prediction model from TRL 5 to TRL 7 would require: (1) integration with a real EHR system through a HL7 FHIR-compliant interface; (2) a human-factors study establishing that clinicians can interpret and act on model outputs; (3) a prospective pilot with pre-specified outcome monitoring over a minimum of six months; and (4) safety monitoring protocols to detect unexpected model failures. These steps are achievable within a two-to-three-year research program and represent the most impactful investment the field can make [59,60].

### 5.4. Explainable AI (XAI): A Research Priority for Clinical Adoption

The four reviewers of this manuscript uniformly identified the absence of a dedicated discussion on Explainable Artificial Intelligence (XAI) as a significant gap. This section addresses that observation directly by synthesizing the current state of XAI in clinical AI and outlining the specific methods most applicable to neonatal and perinatal mortality prediction.

#### 5.4.1. Why Explainability Is Non-Negotiable in Neonatal Clinical AI

The clinical adoption of ML and DL models for neonatal mortality prediction faces a fundamental barrier beyond predictive performance: the interpretability gap. Regulatory bodies including the FDA and the European Medicines Agency increasingly require that AI-based clinical decision support tools provide traceable, auditable reasoning alongside their outputs [61]. In neonatal intensive care, where decisions about escalation of care, resource allocation, and parental counseling are made under time pressure, a clinician must be able to understand why a model flags a patient as high-risk to act on that signal. The absence of this capacity is the primary reason why, despite a decade of published predictive models in this domain, none of the 46 studies reviewed has been reported as deployed in an active clinical setting at scale.

#### 5.4.2. Post Hoc Explanation Methods for Tabular Clinical Data

For the structured tabular EHR data that characterizes most reviewed studies, two post hoc explanation frameworks are particularly relevant:

SHAP (SHapley Additive exPlanations) [62] provides theoretically grounded feature attribution based on Shapley values from cooperative game theory. For a given prediction, SHAP computes the marginal contribution of each feature to the deviation from the expected output. Its TreeSHAP implementation runs in polynomial time for tree-based models (Random Forest, XGBoost), making it computationally feasible for real-time clinical deployment. In the neonatal context, SHAP beeswarm plots can visually communicate which clinical variables (e.g., lactic acid, birth weight, oxygen saturation) drive individual risk scores, enabling clinicians to identify actionable intervention targets.

LIME (Local Interpretable Model-agnostic Explanations) [63] generates locally faithful linear surrogate models around individual predictions by perturbing the input and observing output changes. LIME is model-agnostic, making it applicable to any classifier in the ensemble—including logistic regression, decision trees, and neural networks—without requiring access to internal model parameters. It is particularly suitable for deployment in clinical interfaces where a simple ranked list of explanatory features is needed for rapid clinical assessment.

#### 5.4.3. Explanation Methods for Deep Learning and Signal-Based Models

For DL architectures processing cardiotocography (CTG) signals or time-series vital signs, visual explanation methods are required:

Grad-CAM (Gradient-weighted Class Activation Mapping) [64] uses gradients flowing into the final convolutional layer of a CNN to produce a coarse localization map highlighting which signal segments contributed most to the classification. In the perinatal context, Grad-CAM can highlight CTG decelerations or variability patterns that the model associates with adverse outcomes, providing direct visual feedback to obstetricians reviewing fetal monitoring traces.

Attention visualization in LSTM and Transformer-based architecture identifies which time steps in a vital sign sequence most influence the model’s risk prediction, enabling clinicians to correlate model attention with clinically recognized deterioration patterns.

#### 5.4.4. Integration Recommendations for Future Studies

Future research in this domain should treat XAI as a methodological requirement, not an optional enhancement. Concretely, we recommend that studies: (1) report SHAP global importance plots alongside standard performance metrics; (2) evaluate whether SHAP or LIME explanations are consistent with established clinical knowledge as a validity check (“clinical sanity test”); (3) conduct human-factors studies assessing whether explanations improve clinician decision quality and reduce time-to-intervention; and (4) design clinical interfaces that present risk scores and their explanations simultaneously, avoiding the “alert fatigue” associated with unexplained automated flags [61,62].

## 6. Ethical Considerations in the Application of Artificial Intelligence to Neonatal and Perinatal Care

The deployment of AI-based predictive tools in clinical settings—particularly in contexts as sensitive as neonatal and perinatal care—raises fundamental ethical questions that extend beyond technical performance metrics. The reviewed literature addresses these concerns unevenly: while methodological rigor in model development is widely discussed, ethical dimensions such as patient consent, data privacy, algorithmic bias, and equitable access to AI-driven tools remain largely absent from published studies [13,59]. This section examines these dimensions systematically, drawing on the evidence synthesized in this review and on established frameworks in AI ethics and health informatics.

### 6.1. Data Privacy and Patient Consent in Clinical AI

The use of clinical data to train predictive models for neonatal mortality involves inherent tensions between the scientific imperative to generate generalizable knowledge and the fundamental rights of patients to privacy and informational self-determination [59]. In practice, most studies reviewed rely on retrospective administrative datasets or birth registries that were collected for clinical or administrative purposes—not for machine learning research. The use of such data without explicit informed consent raises questions about the scope of the original consent granted at the point of care, particularly when data is subsequently used to train models that may influence clinical decisions affecting future patients [13,60].

Regulatory frameworks address this tension differently across jurisdictions. In high- income countries, HIPAA (United States) and GDPR (European Union) establish strict requirements for de-identification, purpose limitation, and data subject rights, including the right to object to automated decision-making under Article 22 of GDPR [60]. However, de-identification protocols—such as the removal of direct identifiers and the generalization of quasi-identifiers—are not sufficient to eliminate re-identification risk in small neonatal populations where rare clinical combinations may be uniquely identifying [13]. In low- and middle-income countries, formal data protection legislation is often absent or unenforced, leaving patients in the most vulnerable health systems with the least protection over their clinical data [44].

Beyond regulatory compliance, the ethical deployment of AI in neonatal care requires the development of meaningful consent processes adapted to clinical AI contexts. Traditional informed consent models, designed for individual therapeutic interventions, are ill-suited to the diffuse, population-level nature of machine learning research. Emerging frameworks such as broad consent with dynamic opt-out mechanisms, community consultation processes for population-based datasets, and data trusts governed by patient representatives offer more appropriate models for this domain [59,60].

### 6.2. Algorithmic Bias and Inequality in Access to AI Tools

Algorithmic bias in clinical AI refers to systematic errors in model outputs that disproportionately affect specific patient subgroups—typically those underrepresented in training data. In the neonatal mortality domain, this risk is acute and structurally embedded: as documented in Section 3, over 69% of the training datasets used in the reviewed studies come from Europe and North America, meaning that the models developed on this data encode the clinical patterns, variable distributions, and outcome prevalences of high-income populations [43]. When deployed—or extrapolated—to Sub-Saharan African or Latin American contexts, these models may produce systematically poorly calibrated risk scores, potentially leading to both underidentification of at-risk newborns (false negatives with fatal consequences) and overidentification (resource misallocation in already constrained systems) [17,43].

Bias enters predictive models through multiple pathways. At the data collection stage, systematic underrepresentation of rural, indigenous, and low-income populations in clinical registries means that the training distribution does not reflect the full population at risk. At the feature selection stage, the choice of variables is constrained by what is routinely documented in clinical records, which in turn reflects the diagnostic and monitoring capacities of the healthcare system—creating a structural advantage for settings with richer data infrastructure [43]. At the evaluation stage, the use of aggregate performance metrics (accuracy, AUC) conceals subgroup disparities: a model with 90% overall accuracy may perform at 65% for the subgroup—defined by ethnicity, geography, or socioeconomic status—that faces the highest mortality risk [17].

Addressing algorithmic bias requires deliberate methodological choices at every stage of model development. Stratified performance evaluation across demographic and geo- graphic subgroups, fairness-aware training objectives, and participatory model development processes that include clinicians and community representatives from LMIC settings are essential components of ethical AI deployment in this domain [17,43,59].

### 6.3. Inequality in Access to AI-Driven Clinical Tools

Even a technically sound and ethically developed predictive model creates ethical problems if it is accessible only to health systems with the resources to implement it. The concentration of AI development capacity—computational infrastructure, trained data scientists, and research funding—in high-income countries produces a structural pattern in which the populations that bear the greatest burden of neonatal mortality are precisely those least likely to benefit from advances in predictive modeling [44,49].

This access gap operates at multiple levels. At the infrastructure level, many health facilities in LMICs lack the computing resources, internet connectivity, or electronic health record systems necessary to deploy and operate ML-based decision support tools [49]. At the human capital level, the shortage of clinical informaticians and data scientists in low- resource settings limit the capacity to adapt, validate, and maintain externally developed models [43]. At the economic level, proprietary AI tools developed by commercial entities in high-income countries are often priced beyond the reach of public health systems in LMICs, reinforcing existing inequities in healthcare quality [49].

Ethical commitments to health equity require that AI development for neonatal mortality prevention be explicitly oriented toward deployment in the settings where it is most needed. This implies open-source model publication, lightweight model architectures compatible with low-resource computing environments, capacity-building partnerships between high-income research institutions and LMIC health systems, and funding mechanisms—from global health donors and development banks—that prioritize AI health equity as an explicit objective [44,49,59].

### 6.4. Regulatory and Governance Frameworks for Clinical AI

The deployment of AI-based predictive tools in healthcare is increasingly subject to regulatory oversight, and the neonatal mortality prediction domain is not exempt. Three governance frameworks are particularly relevant and should inform the development trajectory of models reviewed in this study.

The U.S. Food and Drug Administration (FDA) Artificial Intelligence/Machine Learning-Based Software as a Medical Device (AI/ML-SaMD) Action Plan establishes that AI-based clinical decision support tools intended to drive clinical decision-making are classified as medical devices and must demonstrate analytical validity, clinical validity, and clinical utility before deployment. The FDA’s proposed framework for “predetermined change control plans” is especially relevant to neonatal AI models, as it would allow models to adapt to new patient populations (e.g., expanding from high-income to LMIC settings) without requiring full re-review, provided that the adaptation process follows pre-specified, validated protocols. No reviewed study has initiated a regulatory submission process under this or any equivalent national framework.

The World Health Organization (WHO) Ethics and Governance of Artificial Intelligence for Health guidance document (2021) establishes six core principles for ethical AI in health: autonomy, well-being, transparency, accountability, inclusiveness, and responsiveness. Applied to neonatal mortality prediction, these principles require that: (1) model outputs augment rather than replace clinical judgment; (2) patients and communities whose data trains the models are meaningfully represented in governance processes; (3) performance disparities across ethnic and geographic subgroups are monitored and reported; and (4) models are designed with the specific epidemiological and resource context of the intended deployment setting in mind. The reviewed corpus addresses none of these principles systematically.

The European Union Artificial Intelligence Act (2024), the world’s first comprehensive legal framework for AI regulation, classifies AI systems used in clinical decision support as “high-risk” AI applications subject to mandatory conformity assessment, technical documentation, transparency requirements, and post-market monitoring. Research groups in EU member states developing neonatal mortality prediction models will be required to comply with these provisions prior to any clinical deployment, representing a significant regulatory bar that reinforces the importance of the TRL-based maturity assessment presented in Section 5.3.

## 7. Conclusions

This paper presents a Structured Literature Review of 46 peer-reviewed studies published between 2018 and 2024, examining the application of Artificial Intelligence (AI) techniques—specifically Machine Learning (ML) and Deep Learning (DL)—to the prevention of prenatal, preterm birth, and neonatal deaths. The review was conducted following Massaro’s protocol [10] across three major databases—Scopus, IEEE Xplore, and Google Scholar—and synthesizes evidence along five analytical dimensions: predictive algorithms, clinical datasets, geographic context, performance metrics, and identified research gaps.

The bibliometric and thematic analysis yields three principal findings. First, machine learning techniques dominate the corpus (71.7% of studies), with Random Forest, Logistic Regression, and Gradient Boosting consistently achieving AUC values between 0.80 and 0.94 on structured clinical data, while deep learning approaches—particularly LSTM and CNN architectures—demonstrate superior performance on sequential signal data such as cardiotocography traces, with reported AUC values above 0.90 in preterm birth prediction [36,56]. Neither paradigm is universally superior: the choice between ML and DL is determined by data modality, available computational infrastructure, and the interpretability requirements of the clinical setting.

Second, a critical and structurally significant geographic imbalance characterizes the corpus. While over 80% of global neonatal and perinatal mortality occurs in low- and middle-income countries (LMICs), fewer than 5% of the training datasets used in reviewed studies originate from Latin America or Sub-Saharan Africa [28]. Europe and North America account for 69.5% of publications and 56.5% of dataset origins, producing models whose epidemiological and infrastructural assumptions do not transfer to the settings where prevention is most urgently needed [38,49]. This geographic gap is the most consequential limitation of the current state of the art.

Third, the field has not yet crossed the threshold from research tools to clinical instruments. Fewer than 15% of reviewed studies include external validation beyond the original institution’s dataset, none report regulatory approval, and integration with hospital information systems or electronic health record platforms remains largely absent from the corpus [13,43]. The absence of standardized outcome definitions, shared benchmark datasets, and reproducible evaluation frameworks prevents cumulative scientific progress and meaningful comparison across studies [58].

Addressing these limitations requires coordinated action across three dimensions. At the data infrastructure level, adoption of HL7/FHIR interoperability standards and the development of open, LMIC-representative clinical datasets are prerequisites for equitable model development. At the methodological level, hybrid ML-DL architectures that combine the interpretability of classical algorithms with the temporal modeling capacity of deep learning, and federated learning frameworks that enable privacy-preserving multi-institutional collaboration, represent the most promising directions for future research [59,60]. At the implementation level, structured co-design processes involving clinicians, data scientists, health informaticians, and regulators are required to produce deployment-ready systems for perinatal care.

Several limitations of this review should be acknowledged. First, the search did not include PubMed/MEDLINE as a primary database. As justified in Section 2, this decision was consistent with Massaro’s protocol for computing research and with Scopus’s substantial overlap with MEDLINE-indexed journals; however, it may have introduced a bias toward algorithmically oriented papers, potentially underrepresenting clinical validation studies. Future systematic reviews on this topic should include PubMed/MEDLINE to ensure comprehensive biomedical coverage. Second, the quality filter applied in Step 4 of the selection process relied on publisher reputation as a proxy for methodological rigor; while this criterion is common in computing-focused SLRs, it may have excluded high-quality studies published in regional or open-access venues, particularly from LMIC research groups. Third, the review covers publications from 2018 to 2024; studies published after December 2024 are not included. Fourth, three of the 46 included studies—[20,39,40]—were hosted on medRxiv at the time of the December 2024 search and had not completed peer review. Their publication type in Table 4 and Table 7 has been corrected from JP (Journal Paper) to PP (Preprint). Future updates of this review should verify whether peer-reviewed published versions have become available and replace or remove these entries accordingly.

The SDG-3 target of reducing neonatal mortality to fewer than 12 deaths per 1000 live births by 2030 remains unmet in the regions where the burden is greatest. AI alone cannot close this gap—but the development of generalizable, equitable, and clinically validated predictive frameworks is a necessary component of any credible prevention strategy. This review provides researchers and healthcare practitioners with a structured foundation for designing the next generation of decision-support tools adapted to the realities of resource-constrained health systems worldwide.

## Figures and Tables

**Table 1:** Complete Boolean search strings by database and query type. All searches were executed in December 2024 using title, abstract, and keyword fields (TITLE-ABS-KEY in Scopus; All Metadata in IEEE Xplore; general search in Google Scholar).

Database	Query	Complete Boolean String
Scopus	A (ML)	TITLE-ABS-KEY ((“Machine Learning” OR “Neural Network” OR “Predictive Model”) AND (“Neonatal Death” OR “Perinatal Death” OR “Preterm Birth” OR “Neonatal Mortality” OR “Prenatal Mortality” OR “Prevention”))
Scopus	B (ML)	TITLE-ABS-KEY ((“Deep Learning” OR “Convolutional Neural Network” OR “LSTM” OR” Recurrent Neural Network”) AND (“Neonatal Death” OR “Perinatal Death” OR “Preterm Birth” OR “Neonatal Mortality” OR “Prenatal Mortality” OR “Prevention”))
IEEE Xplore	A (ML)	(“All Metadata”:“Machine Learning” OR “All Metadata”:“Neural Network” OR “All Metadata”:“Predictive Model”) AND (“All Metadata”:“Neonatal Death” OR “All Metadata”:“Perinatal Death” OR “All Metadata”:“Preterm Birth” OR “All Metadata”:“Neonatal Mortality” OR “All Metadata”:“Prevention”)
IEEE Xplore	B (ML)	(“All Metadata”:“Deep Learning” OR “All Metadata”:“Convolutional Neural Network” OR “All Metadata”:“LSTM”) AND (“All Metadata”:“Neonatal Death” OR “All Metadata”:“Perinatal Death” OR “All Metadata”:“Preterm Birth” OR “All Metadata”:“Neonatal Mortality” OR “All Metadata”:“Prevention”)
Google Scholar	A (ML)	“Machine Learning” OR “Neural Network” OR “Predictive Model” AND “Neonatal Death” OR “Perinatal Death” OR “Preterm Birth” OR “Neonatal Mortality” OR “Prevention”
Google Scholar	B (ML)	“Deep Learning” OR “Convolutional Neural Network” OR “LSTM” AND “Neonatal Death” OR “Perinatal Death” OR “Preterm Birth” OR “Neonatal Mortality” OR “Prevention”

**Table 2:** Steps and search results.

Step	Quantity
0—Initial records identified across Scopus, IEEE Xplore, Google Scholar (ML + DL queries combined)	459
1—Records after deduplication (between databases and between ML/DL queries)	133
1a—Search in Database—ML (ML, Neonatal/Perinatal/Preterm Birth Death/Mortality, Countries)	94
1b—Search in Database—DL (DL, Neonatal/Perinatal/Preterm Birth Death/Mortality, Countries)	39
2—Year of publication filter: select only publications between 2018–2024	79
3—Mortality prevention filter: select only publications related to mortality prevention	63
4—Quality and Credibility filter: select publications from reputable and classified publishers	46

**Table 3:** Publishers.

Code	Publisher
American M.A.	American Medical Association
B.M.J.P.G.	British Medical Journal Publishing Group
BioMed C.	BioMed Central
Cold S.H.L.P	Cold Spring Harbor Laboratory Press
Elsevier	Elsevier
F1000 R.L.	F1000 Research Limited
Hindawi	Hindawi
IEEE	IEEE
Korean A.M.S.	The Korean Academy of Medical Sciences
MDPI	MDPI
MFM	Maternal-Fetal Medicine
Nature P.G.	Nature Publishing Group
Newborn	Newborn
NLM	National Library of Medicine
Oxford U.	Oxford University Press
P.L.S SF	Public Library of Science San Francisco, CA USA
S.A.I	Science and Information (SAI) Organization Limited
Springer	Springer
T&F	Taylor & Francis

**Table 4:** Types of publications.

Code	Type
CP	Conference Paper
JP	Journal Paper
PP	Preprint (medRxiv/not yet peer-reviewed at time of search)

**Table 5:** Topics.

Code	Topic—TIC	Topic—Medicine
ML + PD	Machine Learning	Perinatal Death
ML + PBD	Machine Learning	Preterm Birth Death
ML + ND	Machine Learning	Neonatal Death
DL + PD	Deep Learning	Perinatal Death
DL + PBD	Deep Learning	Preterm Birth Death
DL + ND	Deep Learning	Neonatal Death

**Table 6:** Meta-Data.

Sec.	Meta-Data	Type	Description	Category-RANGE
1	Cons	Numeric	Consecutive associated in article references	11–49
2	Title	Text	Title of publication	No category or rank applicable
3	Type	Text	Type of publication	Journal Paper, Conference Paper
4	Journal	Text	Journal where the publication was made	No category or rank applicable
5	Publisher	Text	Institution/Magazine/Media that made the publication	No category or rank applicable
6	Short name	Text	Short name of the Magazine/Institution/media	No category or rank applicable
7	Year	Numeric	Year of publication	2018–2023
8	Country	Text	Country of publication	No category or rank applicable
9	Continent	Text	Continent of publication	No category or rank applicable
10	Topic–TIC	Text	Prediction tool used to make the model	Machine Learning, Deep Learning
11	SN–Topic–TIC	Text	Short Name of prediction tool	ML, DL
12	Topic–Medicine	Text	Scope of application of the model in fatality prevention	Perinatal Death, Preterm Birth Death, Neonatal Death
13	SN–Topic–Med.	Text	Short Name of scope of application	PD, PBD, ND
14	Cited	Numeric	Number of citations to the publication	1 to ∞
15	Author	Text	Author of the publication	No category or rank applicable
16	Dataset country	Text	Country of the Dataset used for the model	No category or rank applicable
17	Dataset continent	Text	Continent of the Dataset	No category or rank applicable
18	Architecture	Text	ML and DL architecture	No category or rank applicable

**Table 7:** Paper Classifications.

Authors	Reference	Year	SN Type	Cited	ML + ND	ML + PD	ML + PDB	DL + ND	DL + PD	DL + PDB
Tang et al.	[11]	2024	JP	0		✓				
Montazeri et al.	[12]	2024	JP	22					✓	
Koivu and Sairanen	[13]	2020	JP	42		✓				
Rittenhouse et al.	[14]	2019	JP	35			✓			
Cao et al.	[16]	2023	JP	33		✓				
Lee et al.	[17]	2023	JP	2		✓				
Varghese et al.	[18]	2023	JP	1		✓				
Ushida et al.	[19]	2023	JP	1		✓				
Luciana Correia and Beluzo et al.	[20]	2022	PP	1	✓					
Sun et al.	[21]	2022	JP	3			✓			
Valavani et al.	[22]	2022	JP	7			✓			
Shukla et al.	[23]	2022	JP	1	✓					
H. J. Do et al.	[24]	2022	JP	1	✓					
Matsushita et al.	[25]	2022	JP	1			✓			
Wang et al.	[26]	2022	JP	5		✓				
Vishwapriya et al.	[27]	2022	JP	1	✓					
Bogale et al.	[28]	2022	JP	1	✓					
Arabi et al.	[29]	2021	JP	5		✓				
Batista et al.	[30]	2021	JP	3	✓					
Sheikhtaheri et al.	[31]	2021	JP	5	✓					
Hsu et al.	[32]	2021	JP	3	✓					
Rahman et al.	[33]	2021	JP	2		✓				
Begum et al.	[34]	2021	JP	1			✓			
Alvi et al.	[35]	2021	JP	2				✓		
Feng et al.	[36]	2021	JP	5						✓
Mboya et al.	[37]	2021	JP	8	✓					
Mboya et al.	[38]	2020	JP	11	✓					
Alves et al.	[39]	2020	PP	7	✓					
Luciana Correia and Beluzo et al.	[40]	2020	PP	8	✓					
Jaskari et al.	[41]	2020	JP	15		✓				
Bautista et al.	[42]	2020	CP	3		✓				
Ogallo et al.	[43]	2020	CP	6	✓					
Shukla et al.	[44]	2020	JP	22		✓				
Mboya et al.	[45]	2020	JP	12		✓				
Striano et al.	[46]	2020	JP	1				✓		
Lee et al.	[47]	2020	JP	26						✓
Kefi et al.	[48]	2019	JP	8	✓					
Houweling et al.	[49]	2019	JP	40	✓					
Lee et al.	[50]	2019	JP	50						✓
Gao et al.	[51]	2019	JP	39						✓
Khatibi et al.	[52]	2019	JP	18						✓
Hoodbhoy	[53]	2019	JP	5		✓				
Kuhle et al.	[54]	2018	JP	69			✓			
Fergus et al.	[55]	2018	JP	35						✓
Fergus et al.	[56]	2018	JP	48		✓				
Moharir et al.	[57]	2018	CP	44				✓		
Total					13	12	8	3	3	7

The symbol ✓ indicates that the manuscript belongs to this classification.

**Table 8:** ML and DL Approaches by Clinical Scenario—Algorithms.

Clinical Scenario	ML/DL (n)	Primary Algorithms
Neonatal Death	ML: 13/DL: 3	RF, LR, GBM (ML); LSTM, MLP (DL)
Perinatal Death	ML: 12/DL: 3	GBM, XGBoost, Ensemble (ML); CNN, Attention (DL)
Preterm Birth Death	ML: 8/DL: 7	RF, LR (ML); LSTM, CNN (DL)

**Table 9:** ML and DL Approaches by Clinical Scenario—Strengths.

Clinical Scenario	Strengths
Neonatal Death	ML: interpretable, robust to missing data; DL: captures temporal NICU signals
Perinatal Death	ML: handles multivariate EHR; DL: detects complex multimodal interactions
Preterm Birth Death	DL: superior on CTG signals (AUC > 0.90); ML: interpretable on tabular data

**Table 10:** ML and DL Approaches by Clinical Scenario—Limitations.

Clinical Scenario	Limitations
Neonatal Death	ML: class imbalance sensitivity; DL: requires large, labeled datasets
Perinatal Death	ML: misses nonlinear patterns; DL: low interpretability, clinical adoption barriers
Preterm Birth Death	DL: data-intensive, less effective on structured inputs; ML: underperforms on sequential signals

**Table 11:** TRL scale adapted for clinical AI in neonatal and perinatal mortality prediction, with mapping to the reviewed literature.

TRL	Definition (Clinical AI Context)	Evidence Required/Status in Reviewed Corpus
1	Basic research: theoretical foundations of ML/DL applied to clinical outcomes	Algorithm theory; no clinical data. Not applicable in reviewed corpus.
2	Technology concept: ML/DL formulated as clinical prediction problem	Feasibility studies; synthetic or proxy datasets. Not applicable.
3	Proof of concept: model trained and evaluated on a real clinical dataset	Retrospective study on a single institution; standard metrics reported (accuracy, AUC). Approximately 60% of reviewed studies (28/46).
4	Technology validated in laboratory environment: model compared against baselines	Cross-validation, ablation, feature importance analysis on retrospective data. Approximately 30% of reviewed studies (14/46).
5	Technology validated in relevant environment: model evaluated on independent held-out data	Independent test set from same or similar institution; some external validation. Approximately 10% of reviewed studies (4/46); none with fully external cohort.
6	Technology demonstrated in relevant environment: prospective pilot study	Prospective data collection; model outputs surfaced to clinicians in a controlled pilot. Zero reviewed studies.
7	System prototype in operational environment: clinical interface deployed for testing	EHR-integrated prototype; human-factors evaluation; clinician usability testing. Zero reviewed studies.
8	System complete and qualified: regulatory submission or clinical trial	FDA/CE submission; multi-site randomized or controlled trial. Zero reviewed studies.
9	Proven system in operational deployment: routine clinical use	Active deployment in ≥1 hospital; outcome monitoring; post-market surveillance. Zero reviewed studies.

## Data Availability

The bibliometric data analyzed in this study is available upon reasonable request to the corresponding author. No new datasets were created.

## Abbreviations

The following abbreviations are used in this manuscript:

AI	Artificial Intelligence
AUC	Area Under the Curve
CNN	Convolutional Neural Network
CTG	Cardiotocography
DL	Deep Learning
EHR	Electronic Health Record
EMA	European Medicines Agency
FDA	U.S. Food and Drug Administration
FHIR	Fast Healthcare Interoperability Resources
GDPR	General Data Protection Regulation
GBM	Gradient Boosting Machine
HIPAA	Health Insurance Portability and Accountability Act
HL7	Health Level Seven
ICU	Intensive Care Unit
LIME	Local Interpretable Model-agnostic Explanations
LMIC	Low- and Middle-Income Country
LR	Logistic Regression
LSTM	Long Short-Term Memory
ML	Machine Learning
MLP	Multilayer Perceptron
NICU	Neonatal Intensive Care Unit
PAHO	Pan American Health Organization
PRISMA	Preferred Reporting Items for Systematic Reviews and Meta-Analyses
RF	Random Forest
SDG	Sustainable Development Goal
SHAP	SHapley Additive exPlanations
SLR	Structured Literature Review
SVM	Support Vector Machine
TRL	Technology Readiness Level
XAI	Explainable Artificial Intelligence
WHO	World Health Organization
